# Neuronal Gene Targets of NF-κB and Their Dysregulation in Alzheimer's Disease

**DOI:** 10.3389/fnmol.2016.00118

**Published:** 2016-11-09

**Authors:** Wanda M. Snow, Benedict C. Albensi

**Affiliations:** ^1^Division of Neurodegenerative Disorders, St. Boniface Hospital ResearchWinnipeg, MB, Canada; ^2^Department of Pharmacology and Therapeutics, University of ManitobaWinnipeg, MB, Canada

**Keywords:** nuclear factor kappa B, Alzheimer's disease, neuronal gene target, mouse models, memory

## Abstract

Although, better known for its role in inflammation, the transcription factor nuclear factor kappa B (NF-κB) has more recently been implicated in synaptic plasticity, learning, and memory. This has been, in part, to the discovery of its localization not just in glia, cells that are integral to mediating the inflammatory process in the brain, but also neurons. Several effectors of neuronal NF-κB have been identified, including calcium, inflammatory cytokines (i.e., tumor necrosis factor alpha), and the induction of experimental paradigms thought to reflect learning and memory at the cellular level (i.e., long-term potentiation). NF-κB is also activated after learning and memory formation *in vivo*. In turn, activation of NF-κB can elicit either suppression or activation of other genes. Studies are only beginning to elucidate the multitude of neuronal gene targets of NF-κB in the normal brain, but research to date has confirmed targets involved in a wide array of cellular processes, including cell signaling and growth, neurotransmission, redox signaling, and gene regulation. Further, several lines of research confirm dysregulation of NF-κB in Alzheimer's disease (AD), a disorder characterized clinically by a profound deficit in the ability to form new memories. AD-related neuropathology includes the characteristic amyloid beta plaque formation and neurofibrillary tangles. Although, such neuropathological findings have been hypothesized to contribute to memory deficits in AD, research has identified perturbations at the cellular and synaptic level that occur even prior to more gross pathologies, including transcriptional dysregulation. Indeed, synaptic disturbances appear to be a significant correlate of cognitive deficits in AD. Given the more recently identified role for NF-κB in memory and synaptic transmission in the normal brain, the expansive network of gene targets of NF-κB, and its dysregulation in AD, a thorough understanding of NF-κB-related signaling in AD is warranted and may have important implications for uncovering treatments for the disease. This review aims to provide a comprehensive view of our current understanding of the gene targets of this transcription factor in neurons in the intact brain and provide an overview of studies investigating NF-κB signaling, including its downstream targets, in the AD brain as a means of uncovering the basic physiological mechanisms by which memory becomes fragile in the disease.

First described in the context of immunity, NF-κB is gaining acceptance as a key transcriptional regulator of learning and memory. The present review will address NF-κB in the general context of learning and memory as it relates to the literature in AD, the devastating age-related dementia characterized by extracellular amyloid beta (Aβ) plaques, hyperphosphorylated-tau-containing neurofibrillary tangles (NFTs), severe loss of neurons, and cognitive deficits, particularly those related to memory. As we (Snow et al., 2014) and others (Salles et al., 2014; Engelmann and Haenold, 2016) have recently reviewed the role of NF-κB in synaptic plasticity and cognition elsewhere, this topic will not be addressed at length here.

## NF-κB and its activation

**Figure 1:**
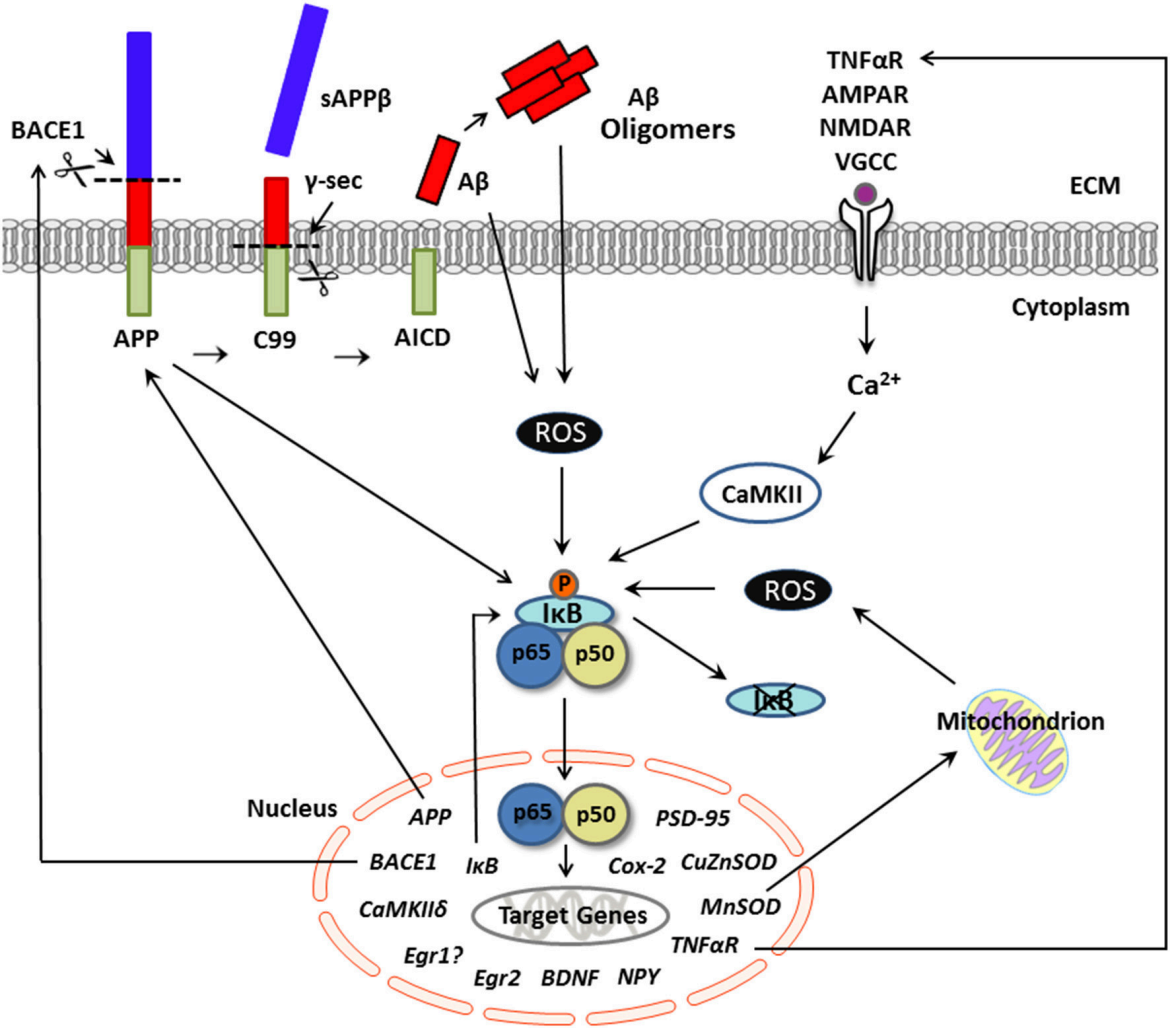
**Putative pathological production of Aβ and NF-κB signaling in neurons**. Schematic representation of activators and neuronal gene targets of NF-κB under conditions of excessive Aβ, as in Alzheimer's disease. The transcription factor NF-κB is activated by several biological agents and thus may serve to integrate various cell signaling pathways in neurons. In the canonical pathway, neuronal stimulation (e.g., by TNFα, calcium, glutamate) elevates intracellular Ca^2+^ levels, which activates IκB kinase and initiates phosphorylation and degradation of IκB. Evidence suggests that this Ca^2+^-mediated activation of NF-κB is CaMKII-dependent. Once in a dimer state (prototypically p65/p50 subunits), NF-κB translocates to the nucleus and binds to the consensus sequence of neuronal gene targets, many of which are involved not only in synaptic plasticity and memory but also amyloidogenic processing. For example, APP appears to serve as both an activator and a gene target of NF-κB in neurons. The production of Aβ occurs via sequential cleavage of APP by BACE1, which yields sAPPβ, followed by cleavage with γ-secretase, which yields an Aβ peptide. Aβ can aggregate into oligomers. Under conditions of supraphysiological/pathological Aβ levels, stimulation of NF-κB appears ROS-dependent and may activate genes involved in the production of Aβ, including APP and/or BACE-1, further exacerbating amyloid dysregulation in AD. Although gene transcription associated with the p65/p50 NF-κB complex (as shown here) can induce downstream gene expression, NF-κB-driven transcription can also downregulate target genes through activation of p50 homodimers, which can repress gene expression. Aβ, amyloid beta; APP, amyloid precursor protein; BACE1, beta-site APP cleaving enzyme 1; CaMKII, calcium-calmodulin kinase II; NF-κB, nuclear factor kappa b; ROS, reactive oxygen species; sAPPβ, secreted amyloid precursor protein beta; TNFα, tumor necrosis factor alpha.

The activation of NF-κB is multifaceted; in an inactive state, this molecule consists of either homo- or heterodimers of various subunit compositions (i.e., p50, p52, p65, which is also called RelA, RelB, and c-Rel) in the cytoplasm, with dimers bound by the inhibitory protein IκB (Zheng et al., 2011). In neurons, the most common composition consists of the p65, p50, and IκBα subunits (Mincheva-Tasheva and Soler, 2013). In the canonical activation pathway, NF-κB becomes activated by a series of neurochemical events that are initiated upon activation of IκB kinase (IKK). IKK activation results in degradation of IκB via phosphorylation (IκBα at Ser^32^ and Ser^36^; IκBβ at Ser^19^ and Ser^23^; Hayden and Ghosh, 2004), and, upon release of IκB, the resulting dimer becomes activated, translocates to the nucleus, and binds to the DNA consensus sequence of its gene targets, where it can either induce or suppresses gene expression (Zhong et al., 2002). One such target of NF-κB transcription is IκB, thereby providing a negative feedback mechanism to tightly regulate NF-κB-dependent gene transcription (Sun et al., 1993; see Figure 1).

Although, activation of NF-κB is classically initiated by IKK activation and IκB degradation, other activation pathways have been identified. For example, NF-κB activation can also occur via an IKK-independent mechanism. Here, tyrosine phosphorylation of IκB results in release of the dimer without IκB degradation (Imbert et al., 1996). Still a third activation pathway has been identified, with IKK becoming activated by NF-κB-inducing kinase, which causes processing of the p100 subunit to the p52 form prior to dimer translocation to the nuclear (Sun, 2011). Upon dimer release, posttranslational modifications can also alter NF-κB activity. For example, protein kinase A (PKA) can induce phosphorylation of the p65 subunit in its active form (i.e., after sequestration from IκB), resulting in enhanced p65 binding at the nucleus (Zhong et al., 1998). However, this pathway can also inhibit gene expression, as PKA-phosphorylated p65 can associate with nuclear RelB, creating a heterodimer that is unable to bind to DNA, thereby reducing NF-κB activation at the nucleus (Jacque et al., 2005). Further specificity of activity can be achieved by the precise location of posttranslational modifications to p65. For example, acetylation of p65 at Lys-122 and 123 results in diminished transcription (Kiernan et al., 2003), whereas acetylation of p65 at Lys-310 results in enhancements (Chen L. F. et al., 2002, 2005). Thus, the activation of cytoplasmic NF-κB is complex and under the regulation of multiple mechanisms via numerous pathways.

## Activators of NF-κB in neurons

Several activators of NF-κB from a broad range of functional categories have been identified in neurons. Of particular relevance to AD is the finding that amyloid beta (Aβ) peptides can induce NF-κB activation in both neurons (see Figure 1) and astrocytes (discussed in detail below).

The cytokine tumor necrosis factor α (TNFα), implicated in the inflammatory response after tissue injury, is among the most well-studied and well-known activators of NF-κB in both neuronal and non-neuronal cells. TNFα is exquisitely linked with NF-κB, as it serves as both an activator (Collart et al., 1990; Beg and Baldwin, 1994) and its receptor as a gene target of NF-κB (Santee and Owen-Schaub, 1996). Further, both TNFα and NF-κB have been shown to regulate long-term potentiation (LTP) (Albensi and Mattson, 2000), a form of synaptic plasticity and a physiological phenomenon thought to underlie learning and memory formation (Bliss and Lomo, 1973; Bliss and Collingridge, 1993).

The neutrophin nerve growth factor (NGF), which supports cellular survival, is capable of activating NF-κB, as measured by increased nuclear immunoreactivity for the p65 subunit, in hippocampal cultured neurons (Bui et al., 2001), peripheral glial cells (i.e., Schwann cells, Carter et al., 1996), and pheochromocytoma (a.k.a. PC12) cells (Wood, 1995; Bui et al., 2001), a cell line used as a neuronal model. In Schwann cells, the effect is mediated through binding of NGF to p75^NPR^, a neurotrophic receptor for which multiple neurotrophic factors (i.e., brain-derived neurotrophic factor (BDNF); neurotrophin-3) have a similar affinity. Neurotrophin-driven NF-κB activation, however, may be specific to NGF, as neither BDNF nor neurotrophin-3 activates NF-κB in Schwann cells (Carter et al., 1996). Ceramide, a lipid found in cell membranes that also possesses cell signaling properties, is capable of eliciting NF-κB activation in many types of cells (Kolesnick and Golde, 1994), including Schwann cells (Carter et al., 1996) and neurons, where it induces cellular reactions similar to that seen with TNFα (Goodman and Mattson, 1996). A 24-h exposure to ceramide or TNFα increases Ca^2+^-current density in cultured hippocampal neurons (Furukawa and Mattson, 1998). Co-application of TNFα with κB decoy DNA prevented increases in Ca^2+^ current, demonstrating the requirement of NF-κB in the observed effect (Furukawa and Mattson, 1998).

NF-κB is, in turn, sensitive to Ca^2+^ signals and becomes activated in response to elevations in intracellular Ca^2+^ (Meffert et al., 2003; Riquelme et al., 2011). For example, high-frequency field stimulation of both hippocampal and cortical neurons, which elevates levels of intracellular Ca^2+^ as well as hydrogen peroxide, a reactive oxygen species (ROS), induces nuclear translocation of p65 NF-κB (Riquelme et al., 2011). Both endogenously present (e.g., after stimulation) and exogenously-applied hydrogen peroxide activate NF-κB via p65 in hippocampal neurons (Riquelme et al., 2011). ROS are well-documented initiators of NF-κB transcriptional regulation in numerous cell types (for review, see Gloire et al., 2006) but not in others (Schoonbroodt and Piette, 2000). The precise mechanisms responsible for the cell specificity of ROS-induced NF-κB activation are unknown. In neurons, however, stimulation-induced NF-κB activation appears to be mediated by both Ca^2+^ and redox signaling.

Various neurotransmitters, as may be expected based on results of stimulation studies, can elicit NF-κB activity. Exposure to N-methyl-D-aspartate (NMDA) induced a Ca^2+^-dependent translocation of p65 NF-κB subunit to the nucleus and enhanced nuclear binding of NF-κB in cultured cortical neurons (Ko et al., 1998). This effect, however, was transient, lasting approximately 30 min (Ko et al., 1998). Excessive stimulation via excitatory neurotransmitters (e.g., glutamate, which binds to the NMDA receptor) is lethal to neurons (Millar et al., 1987). NMDA treatment leads to a rapid (i.e., milliseconds) increase in intracellular Ca^2+^ (Ko et al., 1998). Neurons are exquisitely sensitive to Ca^2+^ levels, and in the face of excessive intracellular calcium, excitotoxicity can result (Randall and Thayer, 1992). Ca^2+^ influx in and of itself is sufficient to induce NF-κB binding activity in cortical neuronal cultures (Ko et al., 1998). Moreover, blockade of NF-κB results in a decreased level of NMDA-associated- neurotoxicity that is dose-dependent in these neurons (Ko et al., 1998), similar to that found in neurons from the hippocampus and cerebellum (Grilli et al., 1996b). In hippocampal neuronal cultures, elevations in both glutamate and Ca^2+^, implicated in excitotoxicity, activated NF-κB through complexes containing p65/p50. Ca^2+^-induced NF-κB activation, however, has been found to be calcium-calmodulin kinase II (CaMKII)-dependent in cultured hippocampal neurons (Meffert et al., 2003).

Although data reveal that induction of NF-κB occurs in conjunction with cellular events known to be neurotoxic (e.g., excitotoxicity), resulting in neuronal death, and that NF-κB inactivation appears neuroprotective, others have noted a neuroprotective effect of NF-κB activation. In mice lacking the p50 subunit, Yu et al. (1999) reported increased vulnerability to excitotoxicity induced by kainic acid injection into the hippocampus. Interestingly, this enhanced susceptibility was also present in the side contralateral to the injection site, whereas the non-injected side exhibited very little evidence of neuronal death in control mice that received kainic acid injections. Thus, NF-κB appears to confer a significant neuroprotective role in the brain.

Interestingly, the nature of the effect exerted by NF-κB as either neuroprotective or neurotoxic may be activator-specific. For example, activation of NF-κB (p65 and p50 subunits) by insulin-like growth factor, which possesses a wide range of biological actions in the CNS, including regulation of neural development, neurogenesis, and synaptogenesis, has been shown to induce neuroprotection in hypothalamic and cerebellar granule neurons (Heck et al., 1999). In contrast, haloperidol, a dopamine D2 receptor antagonist used to treat psychosis, reduced cell survival despite inducing p65 nuclear translocation and increasing NF-κB DNA binding activity in HT22 cells, a clonal mouse hippocampal cell line (Post et al., 1998). These data suggest neurotoxicity associated with NF-κB activation.

Several kinases implicated in learning and memory, including protein kinase A (PKA) (Zhong et al., 1997, 1998), protein kinase C (PKC) (Kim et al., 2010), and CaMKII activate NF-κB (Meffert et al., 2003). PKA phosphorylates the p65 subunit of NF-κB (Lim et al., 2011) and also directly associates with IκB, which, in turn interacts with p65 (Zhong et al., 1997). In turn, PKA is a gene target of NF-κB in neurons (Kaltschmidt et al., 2006). CaMKII is abundant at synapses, whereas other CaMK members (e.g., CaMKIV) are concentrated in nuclei (Meffert et al., 2003).

More recently, studies have implicated NF-κB in maintaining neuronal homeostasis, in which neurons adapt their activity in efforts to preserve the stability of neural networks. Expression of polo-like kinases (PLKs), which are involved in activity-dependent synaptic remodeling via their ability to regulate dendritic spine retraction, can activate NF-κB in hippocampal neurons through the canonical pathway (e.g., involving IκB degradation induced by IKK activation; Mihalas et al., 2013). In p65-deficient cultured neurons, activation of PLKs resulted in significantly greater dendritic spine and glutamate receptor loss relative to activation of PLKs in control neurons, suggesting that NF-κB serves as a counterbalance to prevent overshooting of dendritic spine loss exerted by PLKs in response to high excitatory activity (Mihalas et al., 2013).

## NF-κB in the AD brain

In addition to data demonstrating a role for NF-κB in maintaining several physiological functions (e.g., synaptic plasticity, learning, and memory), alterations to NF-κB are also associated with pathological states, including AD. AD-related neuropathology includes the characteristic Aβ plaque formation and neurofibrillary tangles (NFT). Post-mortem studies of the AD brain generally indicate increased expression and/or activation of NF-κB, particularly in regions preferentially affected in AD. For example, immunoreactivity for the p65 subunit was elevated in several neuronal elements (neurons and their processes, neurofibrillary tangles, dystrophic neurites), particularly in the hippocampus and entorhinal cortex in AD patients relative to control subjects (Terai et al., 1996). Immunoreactivity for p65 was particularly abundant in the cytoplasm of stained neurons, with weaker nuclear staining. Chen et al. (2012) found a robust (50%) increase in p65 protein levels in the frontal cortex of AD patients compared to similar-aged controls. In basal forebrain, the proportion of neurons immunoreactive for p65, either in the cytoplasm, nucleus, or both, was increased in AD (Kitamura et al., 1997). Kitamura et al. (1997) also reported increased p65 levels in particulate, but not cytoplasmic fractions in the temporal cortex, suggesting increased activation of p65-containing dimers in AD. Ferrer et al. (1998) also noted increased p65 immunoreactivity in nuclei of neurons specifically surrounding diffuse (early) plaques in the hippocampus and cortex. In some neurons with evidence of NFT, cytoplasmic immunoreactivity was elevated, suggesting impairment of nuclear translocation in a subset of affected neurons. Further, only neurons, not glia cells, exhibited nuclear immunoreactivity for p65. In nuclear extracts from the temporal lobe of AD patients, NF-κB activation of p65/p50 was detected (Yan et al., 1995). Moreover, immunohistochemical analysis indicated colocalization of p50 and pathological tau and upregulation of p50 in NFT-containing neurons (Yan et al., 1995). Using an antibody raised against epitopes exposed upon IκB degradation, indicative of NF-κB in its active state, Kaltschmidt et al. (1997) documented immunoreactivity of pyramidal neurons and glia surrounding early plaques and within plaques themselves in the limbic cortex and hippocampus, whereas cells distal to plaques were devoid of staining.

The above studies examining proteins of the NF-κB complex are consistent with epigenetic studies examining NF-κB in the AD brain. In addition to studies documenting increased NF-κB at the protein level and evidence of its activation in AD, Rao et al. (2012) reported hypomethylation of the NF-κB promoter CpG region and a concomitant increase in mRNA expression of p65 and p50 subunits in the frontal cortex of those with AD relative to age-matched controls despite hypermethylation of DNA at the global level. Of note is the observation that NF-κB regulates expression of microRNAs, small noncoding single strands of RNA that, in turn, regulate the expression of multiple genes at the post-translational level (Lukiw, 2012). NF-κB-mediated epigenetic factors have also been implicated in disease, including AD (Zhao et al., 2013), a topic that is the subject of recent reviews (Lukiw, 2012; Millan, 2014) and therefore is not addressed at length here.

Although the majority of studies indicate elevations in NF-κB complexes, Kaltschmidt et al. (1999) also reported decreased nuclear localization of p65 in both neurons and glia surrounding mature plaques in cortical tissue in AD cortex relative to that from nondemented controls matched for age, post-mortem delay, and fixation time, suggesting reduced NF-κB activity associated with disease progression in AD. Alternatively, these data suggest aberrations with activation and/ or nuclear translocation of NF-κB-activated dimers in AD. Such an interpretation is consistent with the enhanced cytoplasmic localization of p65-containing dimers found in AD-vulnerable regions reported by Terai et al. (1996).

## Amyloidogenic signaling and NF-κB

Several studies indicate a neurotoxic effect of Aβ, the biological material considered central to AD, in primary embryonic neuron cultures, neuron-like cell lines, and non-neuronal cells, including cell death, increased susceptibility to excitotoxicity, and mitochondrial dysfunction (Mattson, 1997). In human fetal cultured neurons, Aβ treatment had no effect on survival but increased susceptibility to glutamate in a dose-dependent fashion and induced NFT-like pathological changes in the presence of glutamate. No such effects, however, were found in human astrocytes, suggesting a neuron-specific effect of Aβ in human tissue (Mattson et al., 1992). In addition to neurotoxic roles, studies also report a protective effect of Aβ. For example, in young undifferentiated embryonic cultured neurons, Aβ increased cell survival. In more mature differentiated cultured neurons, however, Aβ application was toxic, although this response was only seen with higher doses, suggesting a developmental increased in the resilience to the toxic effects of Aβ (Yankner et al., 1990). Others report trophic effects at higher concentrations in embryonic neurons (Whitson et al., 1989), including enhanced survival (Whitson et al., 1989, 1990) and neuritic outgrowth (Whitson et al., 1990). These data imply that the effects of Aβ are both developmentally regulated, dose-dependent, and cell-specific.

One mechanism by which Aβ may exert its effects (trophic or toxic) appears to be via NF-κB signaling. Several studies confirm regulation of NF-κB by Aβ (Behl et al., 1994; Kaltschmidt et al., 1997, 1999; Bales et al., 1998; Valerio et al., 2006; Chami et al., 2012) that appears to be mediated by ROS (Tanaka et al., 2002). Aβ is capable of permeabilizing cell membranes (Glabe, 2006). In rat cortical cultured neurons and in the neuron-like PC12 cell line, Aβ application induces ROS generation (Behl et al., 1994), an activator of NF-κB (Meffert et al., 2003; Riquelme et al., 2011). Thus, extracellular Aβ could permeabilize the neuronal membrane and result in elevated ROS, which, in turn, would be expected to activate NF-κB (see Figure 1).

The precise direction of the effect of Aβ on NF-κB (e.g., resulting in activation or inactivation), however, appears dose-dependent and cell-specific. For example, Kaltschmidt et al. (1997) found increased immunoreactivity for the activated nuclear form of p65 in both neurons and glia from cerebellar granule cell cultures with low-dose Aβ (0.1–1 μM), whereas this effect was not seen with high-dose treatments. In a subsequent study, Kaltschmidt et al. (1999) further found that activation of NF-κB p65 induced by low-dose (0.1 μM) Aβ protected granule cell cultures from the toxic effects of high (10 μM) Aβ levels. Such data are consistent with the detection of Aβ under physiological conditions and the severe neuronal loss seen in vulnerable regions of the AD brain, where Aβ is elevated.

In contrast to the work of Kaltschmidt et al. Chami et al. (2012) confirmed NF-κB activation with Aβ but only at high (supraphysiological) levels in human embryonic kidney 293 (HEK293) cells. Bales et al. (1998) reported reduced levels of constitutive p-65-mediated NF-κB activation with Aβ (25 or 50 μM) in rat cortical embryonic neurons, an effect dependent upon upregulation of IκB levels tethering cytoplasmic NF-κB. These data are in line with post-mortem studies indicating reduced NF-κB p65 activation surrounding Aβ plaques (Kaltschmidt et al., 1999). In glia cultures, however, NF-κB was activated in response to Aβ treatment and resulted in increased expression of inflammatory cytokines interleukin-1 beta and interleukin-6, both downstream targets of NF-κB in glia (Aisen and Davis, 1994).

In addition to dose and cell-type differences, age appears to be a factor in response to Aβ. In adult cortical neuron cultures, Aβ_42_ (10 μM) treatment reduced the magnitude of p50 nuclear translocation in neurons from middle-aged (11 month) rats, with no effect of Aβ_42_ in neurons from old (24 month) rats (Patel and Brewer, 2008). Co-application of Aβ_42_ with TNF, however, significantly increased translocation of the p50 subunit in both age groups (Patel and Brewer, 2008). This finding may have particular relevance to the hippocampus in AD, a site of neurogenesis in the human brain, and therefore, a site in which neurons of varying ages reside (Eriksson et al., 1998).

In contrast to the aforementioned reports of hindered NF-κB nuclear translocation in the presence of Aβ (Bales et al., 1998; Patel and Brewer, 2008), Valerio et al. (2006) found that Aβ induced nuclear translocation specifically of p65/p50 dimers, whereas no such affect was found with c-Rel-containing dimers in a neuroblastoma cell line. Thus, dimer specificity may impart another element of regulation by which Aβ may exert differential effects. Furthermore, Aβ induced cell death, cytochrome c release, and downregulation of antiapoptotic Bcl-XL, indicative of pro-apoptotic mechanisms, and also resulted in increased intraneuronal levels of Aβ_42_. These effects were prevented by blockade of NF-κB (Valerio et al., 2006). These data are consistent with a pro-apoptotic role for p65/p50 dimers and a neuroprotective role for dimers containing c-Rel (Pizzi et al., 2002). Similar data were obtained in mouse cortical embryonic cultures, whereby Aβ application (5 μM) was associated with increased cell death and activation of p50/p65 NF-κB dimers, whereas stimulation of c-Rel-containing NF-κB complex via metabotropic glutamate receptors type 5 protected neurons from Aβ toxicity (Pizzi et al., 2005).

Upon activation, NF-κB can, in turn, regulate Aβ levels, with studies reporting p65/p50-mediated increases in Aβ in NT2N human neurons derived from a teratocarcinoma cell line (Valerio et al., 2006). The amyloid precursor protein (APP) is a neuronal gene target of NF-κB (Grilli et al., 1995, 1996a). Thus, a logical hypothesis would be that NF-κB activation results in increased expression of APP, the proteolytic cleavage of which produces Aβ. Under physiological conditions, NF-κB appears to negatively regulate both APP and Aβ (Valerio et al., 2006; Chami et al., 2012). In pathological conditions (e.g., overproduction of APP and high Aβ levels), however, NF-κB activation upregulates promotor activity of APP and the β-site APP cleaving enzyme (BACE1), responsible for Aβ peptide production from APP cleavage, resulting in elevated Aβ levels (Chami et al., 2012). In turn, APP has been shown to positively regulate NF-κB, as diminished APP results in a robust decrease in NF-κB activation, and conversely, APP overexpression markedly increases NF-κB activity (Chami et al., 2012).

Although the aforementioned studies suggest that NF-κB-mediated elevations in APP production may result in pathological elevations in Aβ, others report no effect of NF-κB activation or inactivation on APP levels, but, rather point to alterations in BACE1 expression, a recognized gene target of NF-κB (Yang et al., 1998; Chen et al., 2012), as a key modulator of NF-κB-associated increases in Aβ. The promoter for the *BACE1* gene contains functional NF-κB binding elements (Chen et al., 2012) that are highly conserved across species (Bourne et al., 2007). In AD frontal cortex, p65 protein levels and BACE1 mRNA are upregulated compared to similar age controls (Chen et al., 2012). In neuronal cell lines, p65 upregulated β-secretase cleavage and production of Aβ; downregulation of p65 with non-steroidal anti-inflammatory agents (NSAIDs) inhibited TNF-mediated BACE1 elevations. No difference in APP protein levels were detected in response to p65, thus elevated Aβ production occurred via enhanced APP cleavage by upregulation of BACE1 (Chen et al., 2012). In other studies, however, activation of NF-κB has been shown to suppress BACE1 expression in neuronal cell lines, specifically through binding of p52/c-Rel dimers. After Aβ treatment, however, NF-κB activation was associated with increased BACE1 protein levels (Bourne et al., 2007). Whether upregulation of this secretase resulted in further increases in Aβ, however, was not examined. Although, the literature is ambiguous regarding the precise mechanisms by which APP and its cleavage into Aβ become neuropathological in AD, data nonetheless indicate that conditions in which amyloidogenic signaling is awry appear to self-propagate further amyloidogenic dysregulation and that NF-κB appears to be centrally involved in this process.

## Tau pathology and NF-κB

In addition to amyloidogenic disturbances, alterations in tau, resulting in NFT formation, constitute the other pathological hallmark of the AD brain. In comparison to mechanisms of Aβ production, the association between NF-κB and tau pathology in AD has received much less attention. Tau is a microtubule-associated protein preferentially expressed in neurons that provides stability to microtubules, primarily through phosphorylation; the hyperphosphorylation of tau is linked to the formation of paired helical filamentous tau, aggregation, and consequently intracellular NFT formation (Šimic et al., 2016). The driving hypothesis in AD for decades has been the “amyloid cascade hypothesis”, which argues that amyloid perturbations precede tau pathologies in AD (Hardy and Allsop, 1991; Hardy and Higgins, 1992). In the 3xTg model, the only model to demonstrate both Aβ plaques and NFTs, plaque deposition occurs prior to NFT development, consistent with this hypothesis (Oddo et al., 2003a). Further, in a 3-D culture system of AD using human neural progenitor cells with mutations in genes involved in amyloidogenic signaling (e.g., APP, presenilin 1 (PS1)), tau pathology results (Choi et al., 2014). Other lines of data, however, refute the amyloid cascade hypothesis. For example, animal models of AD that overexpress APP and exhibit intensive Aβ pathology lack NFT pathology (e.g., CRND8; Chishti et al., 2001). Moreover, glycation of paired-helical filamentous tau in neuroblastoma cells results in elevations in APP and Aβ through NF-κB-dependent pathways (Yan et al., 1995), suggesting a reciprocal relationship between amyloidogenic and tau disturbances that may be mediated through NF-κB. Additional research is needed to explore this connection in AD.

## CREB and NF-κB

Among the most well-studied of the transcription factors implicated in synaptic plasticity, learning and memory is cAMP response element-binding protein (CREB). Decades of research have established a robust role for CREB in memory across species (see Kandel, 2012; Alberini and Kandel, 2014 for reviews). Although not considered a *bona fide* gene target of NF-κB in neurons, several lines of evidence confirm cross-talk between CREB and NF-κB, in line with a vital role for both transcriptional regulators in learning and memory. For example, in catecholaminergic locus coeruleus-like cell line neurons, CREB silencing with siRNA inhibited angiotensin-II mediated activation, nuclear translocation, and DNA binding of p65-containing NF-κB dimers (Haack et al., 2013). Co-immunoprecipitation experiments confirmed no direct association between CREB and NF-κB; however the co-activator CREB-binding protein (CBP), a nuclear integrator that can orchestrate multiple signaling cascades within a cell, associated with CREB and with NF-κB (Haack et al., 2013). Further, blockade of CBP prevented DNA binding of both CREB and NF-κB p65 (Haack et al., 2013). These data suggest CBP acts as a molecular bridge whereby these two transcriptional regulators can act in concert in neurons in the absence of a direct physical association.

In accordance with this, Haack et al. (2013) found that CREB and the p65 subunit of NF-kB associate with different regions of CBP, which would confer CBP with the ability to integrate CREB- and NF-κB-mediated signals in tandem. Others, however, report that PKA-induced phosphorylation of p65 at Ser276 and CREB at Ser133 both involve association with the same KIX region of CBP in nonneuronal cells, including Jurkat T and COS cells (Zhong et al., 1998; Sheppard et al., 1999). In the nucleus, CBP is present in limited supply (Kwok et al., 1994; Shenkar and Abraham, 1999). These data argue for a possible antagonistic relationship between CREB and NF-κB, with competitive binding of each to CBP and indicate another layer of transcriptional regulation within the nucleus (Wadgaonkar et al., 1999). CBP-CREB and CBP-NF-κB p65 binding was strengthened after lung injury *in vivo* and was associated with similar upregulation of gene targets of CREB and NF-κB, with evidence of competitive binding of each to limited CBP (Shenkar et al., 2001). Negative regulation of NF-κB p65-mediated transactivation and gene transcription via PKA-mediated CREB signaling was reported in cultured human monocytic and endothelial cells (Ollivier et al., 1996). In the brain, reductions in neuronal NF-κB inhibit activation of CREB through pathways involving PKA (Kaltschmidt et al., 2006), known to activate CREB by Ser133 phosphorylation (Walton and Dragunow, 2000). In healthy neurons (Yalcin et al., 2003) and in pathological conditions (Lanzillotta et al., 2010), p65 interacts with CBP. Moreover, Aβ treatment results in downregulation of CBP that is rescued by TNFα pretreatment, an effect that appears specific to neurons in the brain and is reliant on TNFα-induced activation of NF-κB (Saha et al., 2009). Such scenarios indicate a complex transcriptional network involving CREB, NF-κB, and CBP and suggest CBP is another sensitive regulator of synapse—nucleus signaling that may be involved in activity-dependent transcription and plasticity.

## CREB in AD

As discussed above, evidence suggests that CREB and NF-κB, upon activation by a variety of stimuli (injury/experience), operate in a coordinated fashion to regulate transcription of several target genes in neurons. Not surprisingly, then, dysregulation of these transcription factors in neurodegenerative disease, such as AD, could be expected to disturb multiple cellular processes that could cumulatively account for cognitive and neurobehavioural disturbances in AD.

Several lines of research implicate altered CREB signaling in AD. For example, in post-mortem AD brain tissue, CREB activation is downregulated in the hippocampus, a region susceptible to AD-related neuropathology. Under experimental conditions, application of Aβ_42_ inhibits CREB activation (Tong et al., 2001; Arunsundar et al., 2015) through decreased synaptic NMDA-receptor density and corresponding reduced NMDA-mediated current, as NMDA-receptor activation phosphorylates CREB at Ser133 (Shaywitz and Greenberg, 1999). Under *in vivo* conditions, elevated amyloid contributes to reduced CREB activation, as TgCRND8 mice, characterized by early, excessive brain amyloid, also exhibit diminished activation of CREB (Yiu et al., 2011). To further understand the potential role of CREB signaling in AD-mediated brain pathology and memory impairments and its utility as a therapeutic target, Yiu et al. (2011) conducted a well-designed series of experiments in the TgCRND8 strain. Elevating CREB activation specifically in the dorsal hippocampus using viral vectors rescued not only spatial memory impairments but also several cellular parameters, including dendritic stunting and reduced spine density in TgCRND8 AD mice, suggesting the utility of CREB to promote both morphological and neurobehavioral recovery in AD. Interestingly, CREB is also present in mitochondria (Cammarota et al., 1999) which exhibit morphological and functional impairments in AD (Cabezas-Opazo et al., 2015) and in mouse models of the disease (Chowdhury et al., 2015). In mitochondria, CREB disruption impairs mitochondrial gene expression, respiration (Lee et al., 2005), and biogenesis (Sheng et al., 2012) as well as neuronal survival (Lee et al., 2005). Further, phosphorylation of mitochondrial CREB increased after one trial of inhibitory avoidance training, suggestive of an involvement of mitochondrial CREB in activity-dependent neural changes (Bevilaqua et al., 1999). Given these data, and the finding that synaptic deficits are the strongest correlates of cognitive dysfunction in AD (Terry et al., 1991), some have argued that therapeutic approaches directed at CREB signaling pathways constitute our best chance for treating AD (Teich et al., 2015), given the diverse role of CREB in neuronal function and positive neurobehavioral ramifications of its upregulation in AD models (De Felice et al., 2007; Saura and Valero, 2011; Teich et al., 2015).

## Manganese superoxide dismutase (MnSOD)

An inducible antioxidant enzyme localized exclusively within the mitochondria, MnSOD (a.k.a. SOD2) acts to scavenge free radicals produced during cellular energy production, namely oxidative phosphorylation (Sompol et al., 2008). MnSOD reduces O_2_ conversion to H_2_O_2_ in the mitochondrial matrix. Compared to other organs, the brain is considered particularly sensitive to oxidative stress, as are neurons specifically. This is largely due to high oxygen consumption levels coupled with a limited presence of scavenger molecules relative to other systems. In lymphocytes of AD patients, MnSOD mRNA levels are elevated, whereas the total antioxidant status/trapping capacity is decreased in plasma (De Leo et al., 1998). Thus, overexpression of MnSOD may be a homeostatic response to mitochondrial perturbations and changes in redox signaling in AD.

In a study by Marcus et al. (2006), MnSOD protein levels, as detected by immunohistochemistry, was found to be highly elevated in multiple subfields (CA1, CA2/3, and CA4) of the hippocampus in AD relative to age-matched non-AD controls; however the lowest increase was seen in the CA1, where AD-related upregulation of MnSOD was significantly lower. Within-group immunoreactivity was similar in controls, whereas immunoreactivity was lowest in the CA1 region relative to other subfields in the AD group. Age was positively correlated with MnSOD immunoreactivity in the control but not the AD group, indicating that age-dependent upregulation of antioxidant-scavenging capacity via MnSOD is diminished in the AD hippocampus. Of note is the finding of extensive pathology specifically in the CA1 subfield post-mortem in the AD hippocampus (Marcus et al., 2006). In contrast, Kairane et al. (2014) found diminished MnSOD activity in cortical mitochondria from AD patients. Elevated mRNA may be a homeostatic response to decreased activity of the enzyme such that functional levels overall remain sufficient.

In further support of a relationship between redox capacity and AD, mice heterozygote for MnSOD (resulting in oxidative stress) crossed with transgenic mice Tg19959 mice overexpressing human amyloid precursor protein (hAPP) displayed elevated Aβ levels as well as increased Aβ plaque deposition relative to Tg19959 mice alone (Li et al., 2004). Conversely, crossing this same strain with MnSOD-overexpressing mice decreased plaque load, rescued spatial memory deficits, and restored synaptophysin levels, indicating synaptogenesis and reversal of AD-related synapse loss without affecting Aβ levels (Dumont et al., 2009). Similar effects were reported by Massaad et al. (2009) using the Tg2576 strain, also possessing hAPP carrying the Swedish mutation (K670N:M671L). In this report, memory was improved, as was the extent of plaque deposition, without affecting total Aβ levels. The ratio of Aβ_42_ to Aβ_40_ was altered such that higher levels of the less toxic Aβ_40_ were found in AD mice with enhanced mitochondrial antioxidant capacity.

Despite these positive findings in mice in which MnSOD is manipulated genetically and the reported alterations to MnSOD in the AD brain, human genetic studies have failed to find a significant association between polymorphisms affecting MnSOD and AD risk (Ventriglia et al., 2005; Paz-Y-Miño et al., 2015; de Mendonca et al., 2016). Thus, AD-related disturbances in MnSOD likely occur secondary to other disturbances or as a result of environmental factors. In accordance with this, in APP/PS1 double transgenic mice overexpressing hAPP and PS1, a genetic candidate for familial AD, social isolation compounded AD-related memory impairments, increased MnSOD levels (indicative of an adaptive response to oxidative stress), and increased the ratio of Aβ_42_ to Aβ_40_ as compared to non-isolated mice (Huang et al., 2011). Chronic exercise (treadmill running for 20 weeks) upregulated MSOD activity, decreased Aβ_42_ levels, and improved long-term memory in the passive avoidance test in APP/PS1 Tg mice relative to sedentary mice, whereas no such differences were found for wild-type (WT) mice (Bo et al., 2014). Such data suggest that non-genetic manipulations that alter MnSOD levels can affect both cognitive and amyloidogenic aspects of AD. In support of MnSOD alterations as occurring secondary to other disturbances, APP/PS1 had no decrease in MnSOD protein levels at varying ages (2–14 mos) in either WT (APP/PS1 heterozygotes) or Tg (APP/PS1 homozygotes) lines, whereas MnSOD activity was decreased (Anantharaman et al., 2006). Nitrotyrosine was increased in Tg mice as compared to WT controls and contributed to the decreased enzymatic activation. These authors suggest Aβ-induced elevations in nitration negatively affect MnSOD activity (Anantharaman et al., 2006).

Experiments *in vitro* in the APP/PS1 strain reveal elevated levels of both MnSOD protein and activation in young cultured neurons vs. neurons from WT mice and continued elevations in both level and activity of MnSOD in neurons from both strains with days *in vitro* (DIV), although this developmental increase in neurons was to a lesser degree in APP/PS1 cultures. These data indicate early oxidative stress in young neurons as well as decreased mitochondrial antioxidant capacity in mature neurons in this AD model (Sompol et al., 2008), consistent with reports in humans (Marcus et al., 2006).

Several lines of research confirm involvement of MnSOD-mediated redox regulation in AD and suggest that targeting this enzyme may represent a useful therapeutic approach for the disease. More work is needed, however, as little is known about this key antioxidant enzyme or the effects of alterations to it in the presence of another key pathological feature of AD in addition to Aβ plaques, namely NFTs, as in the 3xTg mouse (Oddo et al., 2003b). Perturbations to MnSOD would be expected to arise as a consequence of alterations to signaling pathways upstream, including NF-κB. The association between NF-κB and MnSOD in AD-like conditions has only been investigated in one study examining the modulatory effects of astrocytes on Aβ-mediated neurotoxicity (Aguirre-Rueda et al., 2015). Here, application of Aβ_42_ (5 μM over 24 h) was neurotoxic in neuron-only cultures, resulting in a 50% decrease in cell viability relative to neurons treated with Aβ_40−1_ (control treatment). This effect was abolished in neurons co-cultured with astrocytes. Although no decrease in cell viability was observed in astrocytes, Aβ_42_ application upregulated p65 in the nucleus in conjunction with decreased IκB cytoplasmic protein levels, indicative of increased activation of NF-κB complex with Aβ. In accordance, MnSOD protein levels were increased (Aguirre-Rueda et al., 2015). The presence of a similar relationship between Aβ, NF-κB and MnSOD in neurons has not been directly confirmed in either *in vivo* or *in vitro* models of AD in which Aβ is in excess.

## Copper zinc superoxide dismutase (CuZnSOD)

Another key antioxidant molecule, CuZnSOD (a.k.a. SOD1), is a superoxide scavenging enzyme located more diffusely in the cytoplasm in contrast to the strictly mitochondrial localization of MnSOD. This enzyme has been shown to be a downstream gene target of p65-containing NF-κB complex in a neuronal cell line (Rojo et al., 2004) and has also been implicated in AD. Recently, the rs2070424 polymorphism of the *SOD1* gene was found to be associated with protection from AD (Spisak et al., 2014). Decreased CuZnSOD levels were found in peripheral blood mononuclear cells in patients with mild cognitive impairment, a prodrome to AD, and in the cortex of young 3xTg male mice, suggesting early dysregulation in antioxidant capacity in the AD-like brain (Mota et al., 2015). In double-transgenic AD mice, chronic melatonin treatment reduced cognitive deficits, Aβ load, and CuZnSOD mRNA expression, presumably in response to decreased oxidative stress over time with melatonin treatment, although the positive behavioral and amyloidogenic effects were shown to occur independently of alterations to antioxidant signaling (O'Neal-Moffitt et al., 2015).

## CaMKII

Considered a fundamental synaptic protein, CaMKII is required for the consolidation of memories from hippocampal-dependent short-term to cortical-dependent long-term storage (Frankland et al., 2001). CaMKII, regulated by the Ca^2+^/calmodulin complex, can undergo autophosphorylation, which contributes to the persistent activation of CaMKII at the synapse; it is this self-perpetuating activity that positions CaMKII as a key substrate in the physical translation of a transient experience (e.g., learning) to a persistent biological change (e.g., in the presence of experience-dependent neural plasticity; Sweatt, 2016). For example, mutations preventing autophosphorylation inhibit LTP and memory formation (Giese et al., 1998). CAMKII is also activated by and binds to L-type voltage-gated Ca^2+^ channels (Hudmon et al., 2005). Thus, Ca^2+^ homeostasis within a neuron, the dysregulation of which has long been hypothesized as a central, unifying feature of AD (Khachaturian, 1994), is intricately linked to CaMKII signaling.

CaMKII exists as a holoenzyme consisting of a combination of 12 subunits that are under the control of four genes; *CaMKII*α, β, γ, and δ. Of the subunits, CaMKIIα is the most abundant in the brain, where it is highly localized to glutamatergic synapses (Hanson and Schulman, 1992). The various subunits display functional differences. For example, CaMKIIγ is involved in activity-dependent transporting of Ca^2+^/calmodulin from the membrane surface to the nucleus, where it initiates CREB transcription (Ma et al., 2014). CaMKIIβ regulates spine dynamics, as it binds to F-actin, regulates actin turnover in dendritic spines, and contributes to maintenance of mature spines (Fink et al., 2003; Okamoto et al., 2007). CaMKIIα KO mice demonstrate impaired spatial learning (Silva et al., 1992a) and hippocampal LTP (Silva et al., 1992b). Less is known regarding CaMKIIδ in the CNS. Recently, Federman et al. (2013) demonstrated that CaMKIIδ is upregulated in the hippocampus specifically after long training times in a novel object recognition task. Only extended training was associated with a persistent memory and increased CaMKIIδ levels in mice. Moreover, they confirmed, as have others (Kassed et al., 2004) that CaMKIIδ is a neuronal gene target of NF-κB in the hippocampus, with data implicating p65 and p50 subunits in this association. CaMKII activates NF-κB (Lilienbaum and Israël, 2003), and Ca^2+^-dependent activation of NF-κB is CaMKII-dependent (Meffert et al., 2003). Interestingly, after activation, subunits within the CaMKII holoenzyme can exchange with those from other CaMKII holoenzymes, including inactivated ones, a process that, in addition to autophosphorylation, also contributes to persistent activation (Stratton et al., 2013). CaMKII, in turn, phosphorylates CREB (Sun et al., 1994). Thus, in addition to its distinct ability to self-regulate, CaMKII is both a neuronal effector and downstream target of key transcription factors implicated in learning, memory, and synaptic function.

In the AD brain, Western blotting studies have found no differences in protein levels of CaMKIIα in homogenates from either the hippocampus or cortex relative to non-demented controls (Amada et al., 2005; Tannenberg et al., 2006). Analysis using immunohistochemical methods however, suggests that negative findings regarding CaMKIIα using Western blotting techniques appear likely due to an upregulation of CaMKIIα specifically in remaining CA1 neurons, which are reduced in absolute numbers in AD (Wang et al., 2005). In pre-translational studies in AD, CaMKII mRNA expression levels vary depending on the specific gene (α, β, γ, and δ) and brain region examined (Liang et al., 2008).

Autophosphorylation of CaMKII, particularly at threonine-286 (T286), is critical for spatial memory formation and NMDA-receptor-mediated LTP at CA1 synapses in the hippocampus (Sweatt, 2016), both of which are aberrant in AD. In agreement, T286 autophosphorylation is decreased in the AD hippocampus (Amada et al., 2005). Others have reported subcellular-specific differences in T286 autophosphorylation, including a downregulation at synapses and within dendrites but an upregulation in somata in both the CA3 and dentate gyrus hippocampal subfields (Reese et al., 2011).

In animal models of the disease, similar dysregulation of CaMKII signaling is found. In the hippocampus of APP/PS1 mice, Ca_v_1.2 and phospshorylated CaMKII were decreased, as were the number of cells immunoreactive for phosphorylated CaMKII and Ca_v_1.2 (Min et al., 2013). Calmodulin levels, as measured by Western blotting, were elevated. Phosphorylation of CREB was also decreased, consistent with reduced CaMKII-mediated activation, as was a downstream target of CREB, BDNF. In hippocampal neuron cultures, Aβ_42_ treatment upregulated Ca_v_1.2 and elevated intracellular Ca^2+^ levels. However, phosphorylation of CaMKII was decreased. Calmodulin levels were unchanged (Min et al., 2013). Thus, in both AD models, although signaling cascades upstream of CaMKII activation were in opposing directions, in both cases, activation of CaMKII was reduced. *In vivo*, this was associated with a corresponding reduction of its downstream targets implicated in synaptic plasticity, learning and memory in the AD-like brain.

In this same strain, Gu et al. (2009) found no change in overall levels of CaMKIIα, β, or phosphorylated CaMKII in the cortex. Subcellular localization, however, was affected, as all forms of CaMKII examined (α, β, or phosphorylated) were elevated in the cytosol and reduced in synaptosomes relative to WTs. CaMKII was also reduced in cultured cortical neurons after Aβ application, similar to findings reported in hippocampal cultures (Min et al., 2013), with a reduction in the density of CaMKII at synapses. Further, the membrane localization of the GluR1 subunit, important for alpha-amino-3-hydroxy-5-methyl-4-isoxazolepropionic acid (AMPA) receptor stabilization, was reduced, as was an AMPA-mediated ionic current. Importantly, a similar effect on AMPA-mediated current resulted after CaMKII knockdown and was prevented by CaMKII overexpression (Gu et al., 2009).

These data highlight the importance of CaMKII dysregulation in receptor destabilization and impaired excitatory synaptic transmission associated with AD. Literature supports the notion that enhancing CaMKII activity can alleviate neuropathological and neurobehavioural features of AD (e.g., Zeng et al., 2010; Logan et al., 2011; Wang et al., 2013). Naturally derived Aβ oligomers, either from cultured transgenic cells that secrete hAPP (Shankar et al., 2007) or from the brains of those with AD (Shankar et al., 2008), reduced spine density and synapses in pyramidal neurons from mouse hippocampal slice cultures in a reversible fashion (Shankar et al., 2007, 2008). No such effect was noted with application of Aβ monomers or intact amyloid dense core plaques; toxic effects were only noted when these plaques were solubilized with formic acid (Shankar et al., 2008). Importantly, studies have demonstrated that the detrimental effects of Aβ on spine formation, an integral component of the synapse, are *reversible* (Shrestha et al., 2006; Shankar et al., 2007). Activation of CaMKII in neurons is sufficient to elicit spine formation, which occurs in an activity-dependent manner (Jourdain et al., 2003), which could be one mechanism (Fitzjohn et al., 2006) to partially account for noted improvements with treatments upregulating CaMKII.

## Postsynaptic density protein-95 (PSD-95)

PSD-95 is an integral protein involved in regulating activity-dependent synaptic plasticity. Residing in the postsynaptic density, PSD-95 is involved in numerous processes including trafficking and anchoring of receptors and ion channels, regulation of spine density, and modulation of neurotransmitter—receptor communication and synaptic strength (Fitzjohn et al., 2006). It can bind to several other synaptic proteins, including NMDA receptors (Niethammer et al., 1996) and neuroligins (Irie et al., 1997; Song et al., 1999). Through its interaction with neuroligins, PSD-95 also contributes to determining the ratio of inhibitory to excitatory synaptic contacts (Shankar et al., 2007). Consistent with a prominent role for PSD-95 in regulating the synapse, mice deficient in PSD-95 exhibit deficits in spatial learning and alterations in LTP (Migaud et al., 1998), long-term depression (Park et al., 2008), and spine density (Vickers et al., 2006).

In a series of experiments, Boersma et al. (2011) identified PSD-95 as a gene target of NF-κB in mouse hippocampal cultured neurons. Here, p65 was present and enriched particularly in dendritic spines of hippocampal pyramidal neurons. Moreover, a loss of p65 significantly reduced protein levels of PSD-95, whereas excitatory synaptic input activated NF-κB, increased spine density, and upregulated PSD-95 mRNA. These data strongly suggest a role for NF-κB in synapse regulation and activity-dependent synaptogenesis that occurs through PSD-95 regulation.

Evidence suggests that PSD-95 may be particularly sensitive to Aβ. In cultured cortical neurons, application of Aβ_40_ selectively downregulated PSD-95, whereas levels of several other synaptic proteins, including CAMKII, growth-associated protein (GAP-43), and AMPA receptor subunit GluR2 were unchanged with treatment. In addition, the level of the presynaptically-localized synapsin was also unaffected. Although AMPA receptor levels were not different after Aβ treatment, the Aβ-induced loss of PSD-95 was associated with reduced AMPA receptor density at the cell surface (Roselli et al., 2005), in accordance with a role for PSD-95 in receptor trafficking. One mechanism by which Aβ may downregulate PSD-95 may be through NF-κB signaling, given that several studies demonstrate NF-κB activation in neurons by Aβ (Kaltschmidt et al., 1997, 1999; Pizzi et al., 2005; Valerio et al., 2006; Chami et al., 2012), with specific NF-κB homodimers capable of suppressing gene transcription upon translocation to the nucleus (Zhong et al., 2002). However, this has not been tested in relation to Aβ-induced repression of PSD-95.

Despite the specificity of Aβ on PSD-95 reported *in vitro*, post-mortem studies provide contradictory findings related to the status of PSD-95 in AD, as reviewed in detail by Savioz et al. (2014), including negative findings as well as increased and decreased levels being reported in the literature. The authors speculate that regional differences in susceptibility and/or differences in disease severity may account for the reported inconsistencies. In contrast, generalized reductions in PSD-95 are reported in studies using various models of AD-like pathology, including amyloidogenic Tg2576 mice and mice expressing human tau. In PS1 mice, however, PSD-95 is upregulated in the frontal cortex (Savioz et al., 2014). In the 5xTg model, in which amyloid pathology is robust and early, Shao et al. (2011) found an age-dependent shift in PSD-95 localization from dendritic to somatic compartments in the hippocampus, confirming an aberrant subcellular distribution of this postsynaptically rich protein in association with AD-like neuropathology.

## Early growth response (Egr) factors

The early growth response family of transcription factors consists of four members (Egr1-4) that have been identified in the CNS and appear to be involved in regulating gene transcription in association with activity-dependent plasticity, learning and memory. Their specific functions in the CNS, however, are not fully elucidated (Poirier et al., 2008). Of these, Egr1 (a.k.a. NGFI-A, Krox24, Zif268) is the most well-studied in terms of its role in brain function. As an inducible immediate-early gene, the product of *Egr1* has been implicated as a key molecular mechanism by which activity-dependent signaling from the postsynaptic receptor can be transmitted to the nucleus to initiate gene transcription required for the induction of learning and memory-associated neural changes. Consistent with its function as an immediate-early gene, biophysical characteristics of Egr1 include fast (millisecond) activation in response to stimuli and transient expression (Veyrac et al., 2014). Egr1 is implicated in synaptic transmission (Jones et al., 2001; Penke et al., 2013), learning (Guzowski et al., 2001), memory consolidation (Jones et al., 2001; Penke et al., 2013; although see Lee et al., 2004) and reconsolidation (Lee et al., 2004), and more recently in the recruitment of new neurons into functional networks after hippocampal neurogenesis (Veyrac et al., 2013). Coincident with these roles, levels of Egr1 protein are higher in brain regions involved in these functions, including the hippocampus, particularly the CA1 region, and cortex relative to other regions (Beckmann and Wilce, 1997).

Studies identify *Egr1* as a probable gene target of NF-κB in neurons. For example, in mice, hippocampal-mediated recognition memory requires *Egr1* expression and is dependent upon NF-κB activation (Zalcman et al., 2015). Further, gene array analysis of hippocampal tissue identified *Egr1* as a memory-associated gene candidate of NF-κB and further reported NF-κB binding sequences in the *Egr1* promoter region (Ahn et al., 2008). In other tissue, Egr1 has been identified as a *bona fide* NF-κB gene target (Carayol et al., 2006), but additional studies are needed to establish this unequivocally in neurons.

Another member of the Egr family, Egr2 (a.k.a. Krox20) has recently been confirmed as a gene target in neurons (Nafez et al., 2015). In mouse hippocampal slices, LTP upregulates Egr2 (Williams et al., 1995) in an NF-κB-dependent manner that requires p50 (Nafez et al., 2015). Studies of the behavioral impact of Egr2 regulation, however, have found a gain of function in Egr-2-conditional KO mice lacking forebrain expression, as reflected by improved motor skill learning and enhanced long-term memory (24 h) in an object recognition task (Poirier et al., 2007). These data are surprising, given observed elevations in Egr2 after experimental paradigms mimicking learning and memory *in vitro* and are in contrast to behavioral studies in mice with mutations affecting Egr1 and Egr3, which result in both memory and LTP impairments (Poirier et al., 2008). In Egr1 mutants, however, only late-phase LTP and long-term memory are impaired (Jones et al., 2001), whereas Egr3 mutations result in more widespread impairments, affecting both early- and late-phase LTP and short- and long-term memory formation (Li et al., 2007). Interestingly, using different models to examine experience-dependent gene expression, Egr2 upregulation in multiple brain regions appeared independent of CREB (Lemberger et al., 2008). Collectively, these data illustrate potent yet disparate involvement of the Egr transcription factor family in several forms of activity-dependent plasticity and memory.

In AD, dysregulation of Egr1 is fairly well-established. In a recent microarray study focusing on the CA1 hippocampal subfield, Egr1 was deemed a central regulator of genes implicated in AD (Acquaah-Mensah and Taylor, 2016). Post-mortem studies consistently confirm upregulation of Egr1 in both the cortex (Shim et al., 2003; Lu et al., 2011; Hendrickx et al., 2013) and hippocampus (MacGibbon et al., 1997; Gómez Ravetti et al., 2010; Lu et al., 2011). Further, Egr1 and tau were colocalized in the AD hippocampus (MacGibbon et al., 1997). Interestingly, Egr1 mediates tau phosphorylation (Lu et al., 2011), an excess of which contributes to the formation of pathological species of tau and subsequent NFT. Thus, data suggest that: (1) Egr1 upregulation may be a contributing factor to NTF development in AD, and (2) strategies that reduce AD-mediated Egr1 overexpression may offer therapeutic benefit for the disease.

Studies using either animal or *in vitro* models also confirm dysregulation of Egr1 in AD, although the direction of the effect differs in these models from the human condition. For example, intracerebroventricular injection of soluble Aβ_42_ oligomers decreased phosphorylation of CREB and reduced mRNA levels of its target gene, BDNF (discussed below), consistent with data in human AD. However, Egr1 was also decreased, in contrast to the literature on AD (Arunsundar et al., 2015).

In mice, hippocampal Egr1 is elevated after hippocampal-dependent learning tasks, including the Morris water maze and inhibitory avoidance training (Bekinschtein et al., 2007; Porte et al., 2008). In Tg2576 and APP751SL mice, characterized by overexpression of hAPP, hippocampal Egr1 was significantly decreased relative to WT controls after MWM training, in which mice were impaired on learning and memory phases. These data are in agreement with studies documenting epigenetic downregulation of Egr1 in neurons by APP (Hendrickx et al., 2013), a process that has shown to be mediated by CREB (Hendrickx et al., 2014), as overexpression of hAPP would be expected to further suppress Egr1 to a pathological degree. Studies thus far, however, have not investigated the relationship between amyloidogenic processes, Egr1 and tau pathology or how NF-κB alterations potentially link these changes in an AD model in which NFTs occur (e.g., 3xTg strain). Further studies are required to understand the disparate data between AD and experimental models regarding Egr1 transcriptional regulation and cognitive impairments associated with AD. Data thus far, however, indicate that in the AD and AD-like hippocampus, Egr-1-associated activity-dependent transcriptional regulation is altered, which would be expected to exert potent and detrimental effects on synaptic function and ultimately cognition.

Regarding Egr2, few studies have examined this transcription factor in AD. In the aforementioned study by MacGibbon (MacGibbon et al., 1997), although immunohistochemical processing did not reveal any obvious changes in Egr2, semi-quantitative analyses were not conducted. We are not aware of any other study investigating potential Egr2 dysregulation in AD, nor have any studies investigated Egr2 in models of the disease. In light of studies demonstrating enhanced cognition in cases of Egr2 deficiency, it would be interesting to evaluate basal levels of this Egr family member in animals exhibiting AD-associated cognitive dysfunction. Our laboratory has confirmed NF-κB-dependent upregulation of Egr2 after LTP in the hippocampus (Nafez et al., 2015). Whether this upregulation persists in the AD-like brain, however, is unknown.

## Cyclo-oxygenase-2 (Cox-2)

An important regulator of the inflammatory response after injury in neurons is COX-2 (a.k.a. prostaglandin H2 synthase), a rate-limiting enzyme that produces prostaglandin synthase via conversion from arachidonic acid. After cerebral ischemia, COX-2 is severely upregulated, specifically near the infarct border, where it contributes to delayed neuronal death (Nogawa et al., 1997). Interestingly, COX-2 is also induced in the hippocampus after high-frequency stimulation (Yamagata et al., 1993), and its blockade impairs LTP (Chen, C. et al., 2002; Cowley et al., 2008) as well as spatial memory acquisition (Rall et al., 2003; Cowley et al., 2008) and retention (Teather et al., 2002), confirming its importance in regulating synaptic plasticity, learning, and memory.

The NF-κB-COX-2 signaling pathway has been investigated in AD. Epidemiological data suggests that NSAIDs as a class confer neuroprotection against AD onset and clinical progression, with purported effects through regulation of COX isoforms (Pasinetti, 1998). In AD, COX-2 expression is elevated in frontal cortex and in neuron-like cell lines treated with Aβ (Pasinetti and Aisen, 1998). NF-κB activation positively regulates COX-2 expression in neurons (Kaltschmidt et al., 2002), and inhibitors of NF-κB, such as aspirin, result in a corresponding decrease in neuronal COX-2 levels (Kaltschmidt et al., 2002). Further, in aging and in AD, NF-κB binding activity of p65/p50 dimers and COX-2 mRNA were highly correlated (*r*^2^ = 0.87; *r* = 0.93) in cortical tissue (Lukiw and Bazan, 1998). Such data argue for therapeutic benefit with measures that reduce NF-κB and COX-2 downstream, for which there has been support in AD models. In Tg2576 AD mice, pharmalogical inhibition of COX-2 with (NSAIDS) alleviated Aβ-induced memory and synaptic plasticity deficits that are characteristic of this model, effects that occurred independent of decreased levels of Aβ oligomers or inflammation (Kotilinek et al., 2008). In APP/PS1 mice crossed with mice overexpressing COX-2, females exhibited deficits in spatial working memory that were resolved with COX-2 inhibition by pharmacological blockade. No such effects were seen in male transgenic mice. In both sexes, COX-2 overexpression did not affect Aβ plaque load (Melnikova et al., 2006), similar to previous reports (Kotilinek et al., 2008). Others (Guzmán et al., 2009) also reported female-specific deleterious effects of pharmacological blockade of COX-2 in normal mice, including impaired spatial memory retention, with no effect in males (Guzmán et al., 2009). The authors speculated that factors such as differences in drug metabolism and intersecting effects between sex hormones (e.g., estradiol) and COX-2 may explain sex-dependent effects of COX-2 inhibition on learning and memory performance.

## BDNF

The neurotrophin BDNF is ubiquitously expressed in the CNS, suggesting it plays a fundamental role in brain function. Accordingly, BDNF has been shown to participate in multiple neural processes, such as energy metabolism, neurogenesis, neuronal differentiation, and activity-dependent synaptic plasticity, learning, and memory (Noble et al., 2011; Lu et al., 2014). BDNF is regulated by neural activity (Lu et al., 2013) and is also a target of key transcriptional regulators in neurons, including CREB (Tao et al., 1998) and NF-κB (Marini et al., 2004), whose activation elevates BDNF expression (Lipsky et al., 2001). Egr1 has also been shown to bind to BDNF, resulting in decreased expression in neural tissue (Hendrickx et al., 2013).

Additionally, BDNF is regulated by APP (Hendrickx et al., 2013), which has also been shown to suppress BDNF levels. Although, studies demonstrate BDNF elevations in response to CREB activation (Tao et al., 1998), others (Hendrickx et al., 2013) have confirmed CREB-induced decreases in BDNF via regulation through APP. Further, BDNF positively regulates processing of APP by favoring non-amyloidogenic pathways (Rohe et al., 2009), suggesting complex regulatory networks modulating BDNF status in the CNS that involve a key protein implicated in AD. Although APP is able to downregulate BDNF (Hendrickx et al., 2013), APP positively regulates NF-κB, an inducer of BDNF expression (Lipsky et al., 2001). Thus, studies indicate multiple pathways that appear to tightly regulate BDNF expression, which is consistent with its divergent roles in brain function.

Both post-mortem and antemortem studies of AD indicate alterations in BDNF expression. Blood serum studies report increased BDNF in early-stage disease (Laske et al., 2006), which may be an initial compensatory response, whereas post-mortem studies indicate severe decreases in brain BDNF levels (as reviewed in Zhang et al., 2012). Early-stage increases may be indicative of an initial compensatory response in the disease, a hypothesis supported by animal and *in vitro* AD models (Diniz and Teixeira, 2011). Given its pleiotropic roles in the CNS, such deficits in levels of this trophic factor would be expected to contribute diffusely to neural dysfunction in AD.

## Neuropeptide Y

First identified as a gene target of NF-κB in mouse neuroblastoma cells (Musso et al., 1997), neuropeptide Y is an orexigenic peptide that plays a key role in regulating feeding behavior, with a more recently identified functional contribution to learning and memory (Borbély et al., 2013). Neuropeptide Y and its receptors, localized extensively throughout the brain, have been implicated in AD, where levels of both are significantly reduced in the hippocampus and cortex (Beal and Martin, 1986). The ramifications of this in the context of AD are unclear, as are the potential contributions of AD-related NF-κB dysfunction to the observed depression of neuropeptide Y. Animal studies, however, document neuroprotective effects of neuropeptide Y against several insults, including Aβ toxicity (Croce et al., 2012, 2013; Angelucci et al., 2014), in some cases, through modulation of a downstream target of NF-κB, BDNF (Croce et al., 2013).

## Conclusions

As can be appreciated from a survey of the literature, our current understanding of the mechanisms by which Aβ influences NF-κB-driven transcription and vice versa is riddled with discrepancies and inconsistencies. These are likely influenced by methodological differences related to the specific tissue, cell, age, and NF-κB-dimer composition under investigation. Moreover, the majority of studies thus far have investigated the subunits that comprise the prototypical p65/p50 dimer, with few studies investigating other NF-κB subunits (e.g., c-Rel, RelB, or p52) in the context of AD. This is an important consideration, given evidence discussed above indicating differential actions of NF-κB based on the subunit composition. Additionally, although several studies confirm a reciprocal relationship between Aβ and NF-κB, there is a paucity of research examining brain NF-κB, its relationship to AD pathology, and its putative therapeutic potential under conditions in which both cardinal neuropathological features of AD are present, specifically in the presence of excess Aβ AND NFTs. Lastly, the majority of studies examining NF-κB in AD have used model systems from embryonic origins or cancerous cell lines. Given that aging is a significant risk factor for AD (Corrada et al., 2010), research investigating NF-κB in AD using aged animal models or cells derived from aged animals as model systems may generate results that are more generalizable to clinical research in the hopes of translating basic findings into efficacious therapies for the disease (Wallace and Howlett, 2016).

The gene targets of NF-κB in neurons are members of diverse functional classes (see http://www.bu.edu/nf-kb/gene-resources/target-genes/ for a comprehensive list of currently known targets organism-wide, care of Dr. Gilmore, Boston University), including other transcription factors, antiapoptotic proteins, neurotrophic factors, synaptic proteins, antioxidant enzymes, and neuropeptides. Thus, alterations to the NF-κB complex, as has been well-documented in the context of AD, would be expected to have pervasive effects on brain function. Therefore, strategies that impact NF-κB would be, in a similar vein, expected to have potentially broad therapeutic value in treating neuronal dysfunction in AD. On the other hand, its far-reaching effects within an organism, including potent roles in not just cognition but immunity, necessitate a detailed understanding of NF-κB signaling in the CNS so that the ramifications of altering NF-κB for therapeutic gain are more fully appreciated.

## Author contributions

WS formulated the concept and wrote the manuscript. BA was involved in formulating the concept and was involved in editing the manuscript.

## Funding

This work was funded by the National Sciences and Engineering Research Council (to BA) Grant number RGPIN/04742-2014, the St. Boniface Hospital Research Foundation (to BA) Grant numbers 1406-3216, 1403-3131, and 1410-3216, Research Manitoba (to WS, BA), and the Alzheimer's Society of Manitoba (to BA). BA is a Research Affiliate at the University of Manitoba's Centre on Aging, a member of the Children's Hospital Research Institute of Manitoba, and the Honorable Douglas Everett, Patricia Everett, and the Royal Canadian Properties Endowment Fund Chair. BA is also the Manitoba Dementia Research Chair.

### Conflict of interest statement

The authors declare that the research was conducted in the absence of any commercial or financial relationships that could be construed as a potential conflict of interest.

## Abbreviations

AMPA: Alpha-amino-3-hydroxy-5-methyl-4-isoxazolepropionic acid
AD: Alzheimer's disease
Aβ: Amyloid beta
APP: Amyloid precursor protein
BACE1: Beta-site APP cleaving enzyme 1
BDNF: Brain-derived neurotrophic factor
CaMKII: Calcium-calmodulin kinase II
CREB: cAMP response element-binding protein
CNS: Central nervous system
CuZnSOD: Copper zinc superoxide dismutase
Cox-2: Cyclo-oxygenase-2
CBP: CREB-binding protein
Egr: Early growth response
GAP-43: Growth-associated protein
hAPP: Human amyloid precursor protein
IKK: IκB kinase
LTP: Long-term potentiation
MnSOD: Manganese superoxide dismutase
NGF: Nerve growth factor
NFT: Neurofibrillary tangle
NMDA: N-methyl-D-aspartate
NSAID: Non-steroidal anti-inflammatory agent
NF-κB: Nuclear factor kappa b
PLKs: Polo-like kinases
PSD-95: Postsynaptic density protein-95
PS1: Presenilin 1
PKA: Protein kinase A
PKC: Protein kinase C
ROS: Reactive oxygen species
sAPPβ: Secreted amyloid precursor protein beta
TNF: Tumor necrosis factor.
